# Combined use of irinotecan and p53 activator enhances growth inhibition of mesothelioma cells

**DOI:** 10.1002/2211-5463.12985

**Published:** 2020-10-05

**Authors:** Bo Han, Hyeon‐Cheol Lee‐Okada, Momoko Ishimine, Hajime Orita, Keiko Nishikawa, Tetsuya Takagaki, Kazunori Kajino, Takehiko Yokomizo, Okio Hino, Toshiyuki Kobayashi

**Affiliations:** ^1^ Department of Molecular Pathogenesis Juntendo University Graduate School of Medicine Tokyo Japan; ^2^ Department of Biochemistry Juntendo University Graduate School of Medicine Tokyo Japan; ^3^ Department of Gastroenterology and Minimally Invasive Surgery Juntendo University Faculty of Medicine Tokyo Japan; ^4^ Department of Pathology and Oncology Juntendo University Faculty of Medicine Tokyo Japan

**Keywords:** carboxylesterase 2, irinotecan, mesothelioma, p53

## Abstract

Malignant mesothelioma (MM) is an aggressive malignant neoplasm which rapidly invades pleural tissues and has a poor prognosis. Here, we explore enhancement of the effect of irinotecan [camptothecin‐11 (CPT‐11)] by the p53‐dependent induction of carboxylesterase 2 (CES2), a CPT‐11‐activating enzyme, in MM. The level of *CES2* mRNA was greatly increased on treatment with nutlin‐3a. A combination of CPT‐11 and nutlin‐3a inhibited the growth of MM cells more effectively than either drug alone. Knocking down *CES2* in MM cells reduced the effect of the drug combination, and its forced expression in MESO4 cells enhanced the growth inhibitory activity of CPT‐11 in the absence of nutlin‐3a. Enhancement of the growth inhibitory activity of CPT‐11 by nutlin‐3a suggests a possible new combinatorial MM chemotherapy regimen.

## Graphical abstract

We found that the growth inhibitory effect of irinotecan (CPT‐11) on mesothelioma (MM) cells was enhanced by nutlin‐3a (a p53 activator). Nutlin‐3a induced carboxylesterase 2 (CES2), a CPT‐11‐activating enzyme. Knockdown and forced expression of *CES2* confirmed its role in the combined efficacy. The compound use of CPT‐11 and p53 activators might be suitable for development into a potential new MM chemotherapy regimen.

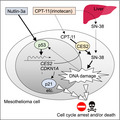

**Abbreviations**
211H: MSTO‐211H cells
ABPP: activity‐based protein profiling
CES2: carboxylesterase 2
CPT‐11: camptothecin‐11
Dox: doxorubicin
FCS: fetal calf serum
H28: NCI‐H28 cells
MESO1: ACC‐MESO‐1 cells
MESO4: ACC‐MESO‐4 cells
MM: malignant mesothelioma
qRT‐PCR: quantitative reverse transcription‐PCR
RNAi: RNA interference
SN‐38: 7‐ethyl‐10‐hydroxycamptothecin
Topo I: topoisomerase I

Malignant mesotheliomas (MMs) are rare fatal malignancies associated with the exposure to asbestos, constituting ~ 0.2% of all newly diagnosed malignancies [1]. MMs originate from mesothelial cells and fall into three main subtypes, epithelioid, sarcomatoid, and biphasic, according to the histological phenotype [2]. MMs of the sarcomatoid subtype have an exceptionally poor prognosis [3]. Most MM patients have unresectable disease, and therefore, different anticancer drug regimens have been tested in clinical trials. However, the results of these have been disappointing [4, 5, 6]. Pemetrexed in combination with cisplatin is currently used as the standard first‐line therapy for unresectable mesothelioma, yielding an overall survival time of 12.1 months [7]. Recently, immunotherapies using immune checkpoint inhibitors have been tried [8, 9]. Although these treatments do provide clinical benefit, MM remains one of the most intractable malignant diseases, and development of more effective therapy is urgently required [10].

Irinotecan (camptothecin‐11; CPT‐11) is a topoisomerase I (Topo I) inhibitor that has been used for the treatment of many types of cancer [11]. It is administered as a prodrug which is hydrolyzed to the active form, 7‐ethyl‐10‐hydroxycamptothecin (SN‐38). The main hydrolyzing enzyme is carboxylesterase 2 (CES2) [12]. It is believed that SN‐38 is generated from CPT‐11 mainly in the liver, but the incomplete hepatic conversion of the prodrug to SN‐38 results in residual CPT‐11 also circulating in the blood [13]. Upregulation of *CES2* gene expression and hence the conversion of CPT‐11 to SN‐38 in the cancer tissue itself may increase drug efficacy. Although CPT‐11 has been tested in MM chemotherapy regimens, its efficacy was limited even in combination with certain other anticancer drugs [13, 14].

Regulation of *CES2* expression by p53 in cancer cell lines was recently reported [15, 16, 17, 18]. p53 is the product of the tumor suppressor gene, *TP53*, and functions in cell cycle arrest, cell death, and differentiation [19]. It is activated by different cellular stresses such as DNA damage, oxidative stress, and hypoxia [20]. The expression of p53 in phenotypically normal cells without excessive stress is downregulated by MDM2, a ubiquitin ligase [20]. *TP53* mutations are found at high frequency in many different cancers [21, 22]. Recent genetic landscape studies of MM revealed that in this tumor, *TP53* mutations were also present, but not at very high frequencies [23, 24]. Thus, the utilization of p53‐dependent mechanisms in novel therapies might be effective for MMs carrying wild‐type *TP53*. One possible method to improve the efficacy of CPT‐11 or other CES2‐dependent prodrugs such as gemcitabine [25] could be the direct activation of p53 in cancer tissues to induce *CES2* locally [16, 17, 18]. The development of chemical p53 activators targeting MDM2 facilitates such a new strategy [26].

In the present study, we investigated the expression of *CES2* in MM cells with wild‐type p53 or loss of p53 expression. We further tested the effect of combining CPT‐11 with the p53 activator, nutlin‐3a [26], on the growth of MM cells.

## Materials and methods

### Cell culture and chemicals

ACC‐MESO‐1 (MESO1) and ACC‐MESO‐4 (MESO4) cells were provided by the RIKEN cell bank (Ibaraki, Japan). MSTO‐211H (211H) and NCI‐H28 (H28) cells were from the American Type Culture Collection (Manassas, VA, USA). All MM cell lines were cultured in RPMI‐1640 medium supplemented with 10% fetal calf serum (FCS), 100 U·mL^−1^ of penicillin, and 100 µg·mL^−1^ of streptomycin, at 37 °C and in 5% CO_2_. Plat‐E cells (COSMO BIO, Hercules, CA, USA) were cultured in Dulbecco's modified Eagle's medium containing 10% FCS, 10 μg·mL^−1^ of blasticidin, 1 μg·mL^−1^ of puromycin, 100 U·mL^−1^ of penicillin, and 100 µg·mL^−1^ of streptomycin at 37 °C in 5% CO_2_. Doxorubicin (Dox), CPT‐11, and nutlin‐3a were purchased from Sigma, Taiho Pharma (Tokyo, Japan), and AdooQ BioScience (Irvine, CA, USA), respectively. SN‐38 and pifithrin‐α (p53 inhibitor) were purchased from Tokyo Chemical Industry (Tokyo, Japan) and Adipogen Life Sciences (Liestal, Switzerland), respectively. DMSO was used as the vehicle for nutlin‐3a and SN‐38. Ethanol (99.5%) was used as the vehicle for pifithrin‐α.

### Cell growth assay

Cell growth was assessed by the XTT assay (Cell Proliferation Kit II; Roche, Basel, Switzerland). Briefly, cell lines were incubated for 24 h after seeding at a density of 2 × 10^3^ cells per well in 96‐well plates. After adding the drug, cells were cultured for another 24 h. after which 50 μL·well^−1^ of the XTT reagent were added and further incubated for 4 h. Measurement of absorbance at 450 nm (reference wavelength of 650 nm) was performed with a microplate reader (Benchmark Plus‐microplate Spectrophotometer; Bio‐Rad, Hercules, CA, USA). Best‐fit IC50 values were calculated with prism 7 (GraphPad Software Inc., San Diego, CA, USA) and compared by an extra sum‐of‐square *F*‐test.

### Western blot analysis

Cells were harvested and lysed in sodium dodecyl sulfate‐polyacrylamide gel electrophoresis (SDS/PAGE) sample buffer (50 mm Tris/HCl, pH 6.8, 2% SDS, and 10% glycerol). Protein concentration was determined by Bio‐Rad DC protein assay (Bio‐Rad). Proteins were separated by SDS/PAGE and transferred onto a polyvinylidene fluoride membrane (Millipore, Carrigtwohill, Ireland). The membrane was blocked with 1% skimmed milk in Tris‐buffered saline containing 0.05% Tween 20 and probed with appropriate antibodies using the Envision system (Dako, Glostrup, Denmark). Signals were developed by Luminata Forte HRP substrate (Millipore) or SuperSignal West Femto Substrate (Thermo Fisher Scientific, Rockford, IL, USA) and then captured by ChemiDoc MP Imaging System (Bio‐Rad). Following primary antibodies were used: anti‐p53 antibody (FL393; Santa Cruz Biotechnology, Santa Cruz, CA, USA), anti‐p21 antibody (H164; Santa Cruz Biotechnology), anti‐CES2 (G5; Santa Cruz Biotechnology), and anti‐GAPDH antibody (6C5; Santa Cruz Biotechnology).

### Quantitative reverse transcription‐PCR

Total RNA from MM cell lines was extracted using the NucleoSpin RNA Kit (TaKaRa, Kusatsu, Japan) according to the manufacturer's protocol. One microgram of total RNA was reverse transcribed with random hexamers using SuperScript III (Thermo Fisher Scientific) to generate cDNA. Quantitative reverse transcription‐PCR (qRT‐PCR) was performed using the Fast SYBR Green Master Mix (Thermo Fisher Scientific). The relative expression of each gene was calculated using the 2^−ΔΔCt^ method. 18S rRNA was used as the reference gene. PCRs were performed in triplicate for all genes. The sequences of the primers used were as follows (forward and reverse, in order) [17]: *CES2*, 5′‐GTAGCACATTTTCAGTGTTCC‐3′ and 5′‐GTAGTTGCCCCCAAAGAA‐3′; *CDKN1A*, 5′‐GATTTCTACCACTCCAAACGCC‐3′ and 5′‐AGAAGATGTAGAGCGGGC‐3′; *NOXA*, 5′‐GCTGGAAGTCGAGTGTGCTA‐3′ and 5′‐CCTGAGCAGAAGAGTTTGGA‐3′; 18S rRNA (reference gene), 5′‐GTAACCCGTTGAACCCCATT‐3′ and 5′‐CCATCCAATCGGTAGTAGCG‐3′.

### Cell death assay

Cells were harvested after 0.25% trypsin‐EDTA treatment and washed with serum‐free medium, and then mixed with an equal volume of 0.4% trypan blue. Viable (nonstained) and nonviable (stained) cells were separately counted in a hemocytometer chamber under a light microscope (CKX41; Olympus, Tokyo, Japan). Cell viability was expressed as a percentage of total number of nonviable cells divided by total number of cells.

### RNA interference

The Silencer Select Predesigned siRNA system (Thermo Fisher Scientific) was employed to suppress *CES2* expression in MESO4 cells. For transfection of siRNAs, the Lipofectamine RNA interference (RNAi) MAX reagent (Thermo Fisher Scientific) was used according to the manufacturer's protocol. Four siRNAs, #s225041 (CESsiRNA#1), #s528 (CESsiRNA#2), #s529 (CESsiRNA#3), and #s529 (CESsiRNA#4), were validated using *CES2*‐overexpressing cells (see below) at a final concentration of 10 nm by qRT‐PCR and two (CESsiRNA#1 and CESsiRNA#3) were selected for further analysis. To test the effect of siRNAs on cell growth, MESO4 cells were first treated with nutlin‐3a (10 µm) for 24 h and then further treated with siRNA (10 nm) and CPT‐11 (5 or 20 µg·mL^−1^) for 24 h. The growth of cells was the quantified using the XTT assay.

### Plasmid construction

Full length human *CES2* cDNA was amplified by PCR using a *CES2* expression plasmid as a template, as described in our previous report [17]. Primers were HCES2F1, 5′‐CCAGATCTCACCATGACTGCTCAGTCCCGCT‐3′ and HCES2R1, 5′‐CCGTCGACCTACAGCTCTGTGTGTCTCT‐3′. Amplified cDNA fragments were digested with *Bgl*II and *Sal*I, and then cloned into the pBabe‐puro vector (Addgene plasmid 1764; Addgene, Watertown, MA, USA). This vector has been modified to contain a *Bgl*II recognition sequence by introduction of synthetic oligonucleotides into the cloning site. Accuracy of the cDNA sequence was controlled by the dideoxy‐sequencing method using an ABI 310 sequencer (Thermo Fisher Scientific). The resulting *CES2* expression vector was designated pBabe‐CES2.

### Establishment of cell lines

Plat‐E cells were transfected with pBabe‐CES2 or empty vector along with an expression plasmid for viral VSVG envelop protein (pCMV‐VSVG) [27] using the FuGENE6 transfection reagent (Promega, Madison, WI, USA) according to the manufacturer's protocol. Forty‐eight hours after transfection, viral supernatants were collected and passed through a 0.45‐μm filter, after which polybrene was added at a final concentration of 8 μg·mL^−1^. MESO4 cells were infected with virus in the supernatant and the selection of stably transduced cells with 1 μg·mL^−1^ of puromycin was started 48 h thereafter. Established cell lines were maintained in the presence of puromycin.

### Activity‐based protein profiling

Cells were homogenized in PBS, and soluble proteins (15 μg in 30 μL of PBS) were incubated with 1 μm of the fluorophosphonate‐rhodamine probe for 30 min at 37 °C. Reactions were then quenched with 4× SDS/PAGE loading buffer (reducing) and separated by SDS/PAGE with 10% (w/v) acrylamide. Conjugated proteins were visualized using a Typhoon FLA 9500 (GE Healthcare Life Sciences, Buckinghamshire, UK). After the detection of conjugated proteins, the gel was subjected to western blot analysis.

## Results

### Effects of the p53 activator and genotoxic agents on MM cell growth and p53 target gene expression

**Fig. 1:**
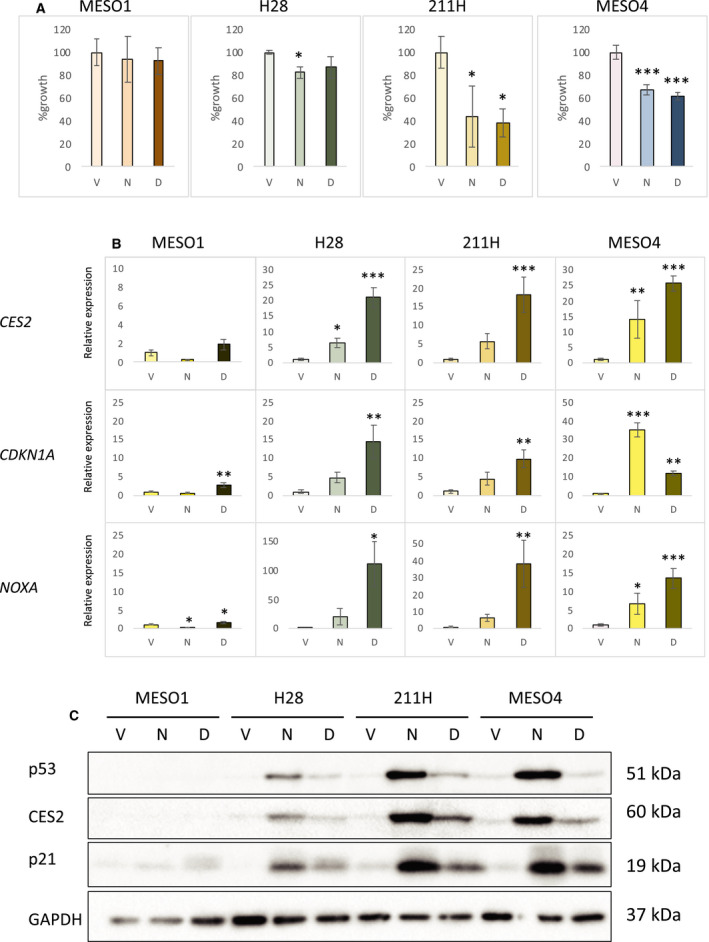
MM cell growth after treatment with nutlin‐3a or Dox. (A) Cell growth analysis. After treatment with vehicle (V), 10 µm of nutlin‐3a (N), or 1 µm of Dox (D) for 24 h, cell growth of four MM cell lines, MESO1, H28, 211H, and MESO4, was analyzed by the XTT assay. The figure shows the mean percent cell growth with SD (*n* = 3), relative to vehicle‐treated control cells. Statistical significance was determined by Dunnett's test. The * and *** represent *P* < 0.05 and *P* < 0.001, respectively, relative to the vehicle‐treated control cells. (B) Induction of *CES2* and p53 target genes in MM cell lines by nutlin‐3a and Dox. MESO1, H28, 211H, and MESO4 cells were treated with DMSO (vehicle; V), 10 µm of nutlin‐3a (N), or 1 µm of Dox (D) for 24 h. The levels of *CES2*, *CDKN1A*, and *NOXA* mRNA were examined by qRT‐PCR analysis. The figure shows the mean expression with SD (*n* = 3), relative to vehicle‐treated control cells. Statistical significance was determined by Dunnett's test. The *, **, and *** represent *P* < 0.05, *P* < 0.01, and *P* < 0.001, respectively, relative to the vehicle‐treated control cells. (C) Expression of p53, CES2, and p21 proteins. Protein samples from MESO1, H28, 211H, and MESO4 cells treated with DMSO (vehicle; V), 10 µm of nutlin‐3a (N), or 1 µm of Dox (D) for 24 h were analyzed by western blotting with anti‐p53, anti‐CES2, anti‐p21, and anti‐GAPDH antibodies.

To analyze the effects of p53 activation and genotoxic stress on cell growth, human MM cell lines expressing wild‐type p53 protein (MESO4, 211H, and H28) [28] or with *TP53* loss (MESO1) [28] were treated with nutlin‐3a (10 µm) or Dox (a nongenotoxic and a genotoxic p53 activator, respectively) for 24 h. We found that nutlin‐3a as well as Dox significantly suppressed the growth of MESO4 and 211H cells and tended to suppress that of H28, although the effect of Dox was not statistically significant in this condition. In contrast, neither drug suppressed the growth of MESO1 to an appreciable extent (Fig. 1A). Next, we determined the expression of the p53 target genes *CDKN1A* [29], *NOXA* [29], and *CES2* [15, 16, 17] in nutlin‐3a‐ or Dox‐treated cells. Using qRT‐PCR, we found that the expression of *CDKN1A*, *NOXA*, and *CES2* was induced in MESO4, 211H, and H28 cells by nutlin‐3a as well as Dox (Fig. 1B). Relative to nutlin‐3a, Dox exerted stronger effects on all three genes, except for *CDKN1A* in MESO4 cells. In MESO1, however, the induction of any of these 3 genes by either drug was minimal, consistent with the loss of p53 function in these cells (Fig. 1B). Western blot analysis confirmed that nutlin‐3a and Dox also remarkably increased their protein levels in MM cells expressing wild‐type p53, but not in MESO1 cells (Fig. 1C). Collectively, these results suggest that the activation of p53 is sufficient to inhibit cell growth of MM cells and that the induction of p53‐regulated genes might be responsible for genotoxic stress‐induced growth inhibition of MM cells.

### Limited effects of CPT‐11 on cell growth and p53 target genes in MESO4 cells

**Fig. 2:**
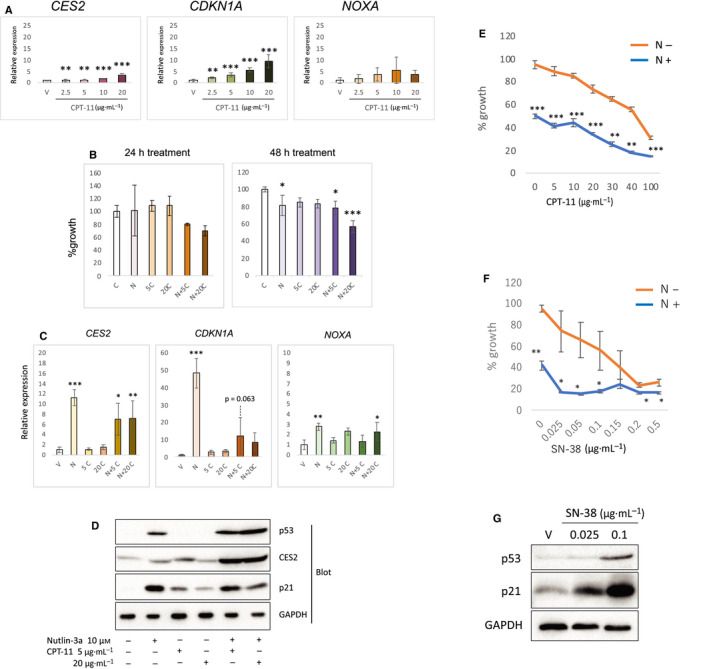
Enhancement of the growth suppressive activity of CPT‐11 by nutlin‐3a in MESO4 cells. (A) Increased expression of *CES2* and p53 target genes by CPT‐11 treatment. The levels of *CES2*, *CDKN1A*, and *NOXA* mRNA in CPT‐11‐treated cells with indicated concentrations were examined by qRT‐PCR. The figure shows the mean expression with SD (*n* = 3), relative to vehicle‐treated control cells. Statistical significance was determined by Williams' test. The ** and *** represent *P* < 0.01 and *P* < 0.001, respectively, relative to the vehicle‐treated control cells. (B) Cell growth analysis. After treatment with drugs for 24 or 48 h, cell growth was analyzed by the XTT assay. The figure shows the mean percent cell growth with SD (*n* = 3), relative to the vehicle‐treated control cells. C = vehicle control, N = nutlin‐3a (10 µm), 5C = 5 µg·mL^−1^ of CPT‐11, 20C = 20 µg·mL^−1^ of CPT‐11. Statistical significance was determined by Dunnett's test. The * and *** represent *P* < 0.05 and *P* < 0.001, respectively, relative to the vehicle‐treated control cells. (C) Expression of *CES2* and p53 target genes. The levels of *CES2*, *CDKN1A*, and *NOXA* mRNA in nutlin‐3a or/and CPT‐11 treated cells were examined by qRT‐PCR. The figure shows the mean with SEM relative to the vehicle‐treated control cells (*n* = 3). C = vehicle control, N = nutlin‐3a, 5C = 5 µg·mL^−1^ of CPT‐11, 20C = 20 µg·mL^−1^ of CPT‐11. The figure shows the mean expression with SD (*n* = 3), relative to vehicle‐treated control cells. Statistical significance was determined by Dunnett's test. The *, **, and *** represent *P* < 0.05, *P* < 0.01, and *P* < 0.001, respectively, relative to the vehicle‐treated control cells. (D) Expression of p53, CES2, and p21 proteins. Cells were treated with drugs as indicated for 48 h. Protein samples were analyzed by western blotting with anti‐p53, anti‐CES2, anti‐p21, and anti‐GAPDH antibodies. (E) Dose response curve of the combined treatment. Cells were treated with various concentrations of CPT‐11 in the presence (N+) or absence (N−) of 10 µm nutlin‐3a for 48 h. The cell growth was analyzed by XTT assay. The figure shows the mean percent cell growth with SEM (*n* = 3), relative to the vehicle‐treated control cells. Statistical significance at each CPT‐11 concentration was determined by Student's *t*‐test. The **, and *** represent *P* < 0.01, and *P* < 0.001, respectively, between N+ and N− cells. (F) MESO4 cells were treated with various concentrations of SN‐38 in the presence (N+) or absence (N−) of 10 µm nutlin‐3a for 48 h. The cell growth was analyzed by XTT assay. The figure shows the mean percent cell growth with SEM (*n* = 3), relative to the vehicle‐treated control cells. Statistical significance at each SN‐38 concentration was determined by Student's *t*‐test. The * and ** represent *P* < 0.05, and *P* < 0.01, respectively, between N+ and N− cells. (G) Expression of p53 and p21 proteins. Cells were treated with SN‐38 at indicated concentrations for 48 h. V; vehicle (DMSO) control. Protein samples were analyzed by western blotting with anti‐p53, anti‐p21, and anti‐GAPDH antibodies.

To examine the efficacy of CPT‐11 for cell growth inhibition and p53 target gene expression, we treated MESO4 cells [in which the induction of *CES2* mRNA was most prominent among the p53‐expressing MM cells tested (Fig. 1B)], with different concentrations of CPT‐11 (2.5–20 µg·mL^−1^). Of the 3 genes examined, *CDKN1A* was induced (Fig. 2A) by CPT‐11 to as similar degree as by Dox (Fig. 1B). However, induction of *NOXA* and *CES2* (Fig. 2A) was not as strong as in Dox‐ or nutlin‐3a‐treated cells (Fig. 1B). At a higher concentration (20 µg·mL^−1^) of CPT‐11, cell growth was inhibited by ~ 15–25% after 48‐h treatment (Fig. 2B,E). These results indicate that the effects of CPT‐11 on cell growth via p53 function are minor in MESO4 cells, probably because of insufficient genotoxicity due to low levels of CES2 enzyme activity.

### Enhancement of the effect of CPT‐11 by p53 activation

We hypothesized that activation of p53 by nutlin‐3a would synergistically enhance the efficacy of CPT‐11 via the induction of *CES2*. To test this possibility, we treated MESO4 cells with CPT‐11 together with nutlin‐3a and assessed cell growth. This combined treatment resulted in more effective inhibition of cell growth in a dose‐dependent manner (Fig. 2B). In this setting, after 24‐h treatment, *CES2* mRNA was increased in cells treated with both drugs relative to CPT‐11 alone, but decreased compared to nutlin‐3a alone (Fig. 2C). We checked p53 and its target protein expression by western blotting. Without p53 accumulation, the level of CES2 and p21 was increased in cells treated with CPT‐11 alone (Fig. 2D). Interestingly, the amount of both proteins was downregulated with higher concentration of CPT‐11 (Fig. 2D). CES2 protein was highly accumulated in cells treated with both drugs as compared with cells treated with either alone (Fig. 2D). Conversely, despite the potent accumulation of p53, the amount of nutlin‐3a‐induced p21 was decreased by simultaneous treatment with CPT‐11, which was similar to the change in the mRNA level (Fig. 2C,D). The IC50 value of CPT‐11 was decreased by nutlin‐3a by 1.6‐fold from 54.85 µg·mL^−1^ [95% confidence interval (CI): 50.82–59.59 µg·mL^−1^] to 34.19 µg·mL^−1^ (95% CI: 28.69–41.7 µg·mL^−1^) in MESO4 cells (Fig. 2E), suggesting that p53 accumulated by nutlin‐3a upregulated *CES2* expression and enhanced the efficacy of CPT‐11 by accelerating its conversion to SN‐38. We further checked if there was a combined efficacy between nutlin‐3a and SN‐38 in MESO4 cells. The growth suppressive effect of SN‐38 was potently enhanced by treatment with nutlin‐3a (Fig. 2F). In contrast to CPT‐11, SN‐38 treatment also induced p53 in MESO4 cells (Fig. 2G). These results taken together suggest that nutlin‐3a upregulates *CES2* expression and accelerates the production of SN‐38, which further contributes to growth suppression of the cells.

### Combined efficacy of nutlin‐3a and CPT‐11, and cell death in MM cells

**Fig. 3:**
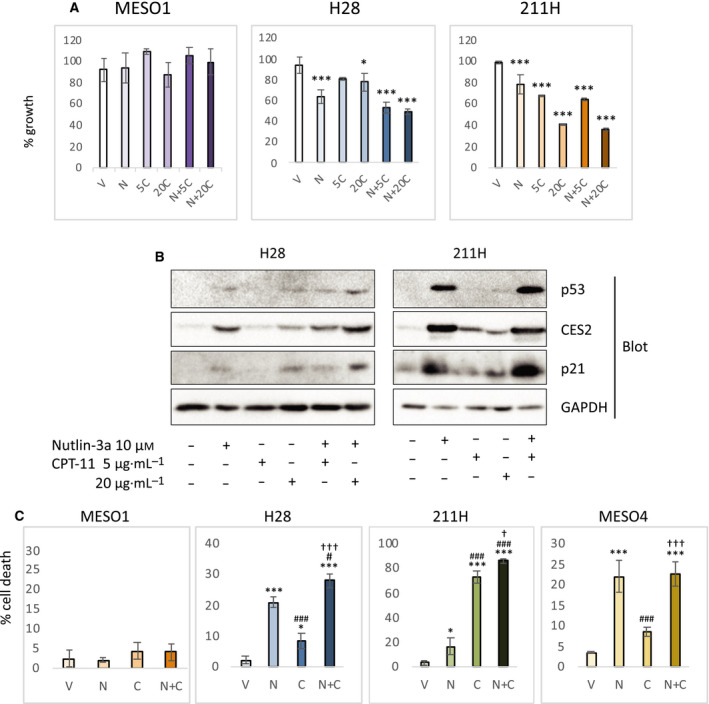
Combined efficacy of nutlin‐3a and CPT‐11 on MM cell lines. (A) Cell growth analysis. After treatment with drugs for 48 h, the growth of MESO1, H28, and 211H was analyzed by the XTT assay. The graphs show the mean percent cell growth with SD (*n* = 3), relative to the vehicle‐treated control cells. C = vehicle control, N = 10 µm of nutlin‐3a, 5C = 5 µg·mL^−1^ of CPT‐11, 20C = 20 µg·mL^−1^ of CPT‐11. Statistical significance was determined by Dunnett's test. The * and *** represent *P* < 0.05 and *P* < 0.001, respectively, relative to the vehicle‐treated control cells. (B) Expression of p53, CES2, and p21 proteins. Protein samples from H28 and 211H treated with indicated drugs for 48 h were analyzed by western blotting with anti‐p53, anti‐CES2, anti‐p21, and anti‐GAPDH antibodies. The sample from 211H cells treated with both nutlin‐3a and 20 µg·mL^−1^ CPT‐11 was not included because massive cell death was occurred (see below) making the protein sample not suitable for analysis. (C) Cell death analysis. After treatment with vehicle (V), 10 µm nutlin‐3a (N), 20 µg·mL^−1^ CPT‐11 (C), or both 10 µm nutlin‐3a and 20 µg·mL^−1^ CPT‐11 (N + C), cells death was assessed by trypan blue exclusion assay. The graphs show mean percent cell death with SD (*n* = 3). Statistical significance was determined by Tukey's test. The * and *** represent *P* < 0.05 and *P* < 0.001, respectively, relative to the vehicle‐treated control cells. The ^#^ and ^###^ represent *P* < 0.05 and *P* < 0.001, respectively, relative to the nutlin‐3a‐treated cells. The ^†^ and ^†††^ represent *P* < 0.05 and *P* < 0.001, respectively, relative to the CPT‐11‐treated cells.

The compound efficacy of nutlin‐3a and CPT‐11 on the cell growth was also observed in H28 cells (Fig. 3A). 211H cells only slightly showed the combined efficacy, possibly due to its high sensitivity to CPT‐11 alone (Fig. 3A,C). On the other hand, the combined efficacy was not observed in MESO1 cells, confirming that the wild‐type p53 is necessary for the enhanced growth suppression by nutlin‐3a and CPT‐11 (Fig. 3A). Unlike in MESO4, p21 was not downregulated by combined use of both drugs in either p53‐wild‐type cell line (Fig. 3B). Compared with cells treated with either drug alone, the combined treatment with nutlin‐3a and CPT‐11 enhanced cell death in H28 and 211H cells (Fig. 3C). In MESO4 cells treated with both drugs, however, the proportion of cell death was comparable to that in the cells treated with nutlin‐3a alone (Fig. 3C), suggesting that cell death and other growth inhibitory mechanisms, such as cell cycle arrest, were both promoted in the combined efficacy of two drugs. As expected from the cell growth assay (Fig. 3A), MESO1 cells exhibited limited cell death upon any single or combined treatment with nutlin‐3a and CPT‐11.

### Involvement of *CES2* in the enhanced growth inhibition resulting from the combination of nutlin‐3a and CPT‐11

**Fig. 4:**
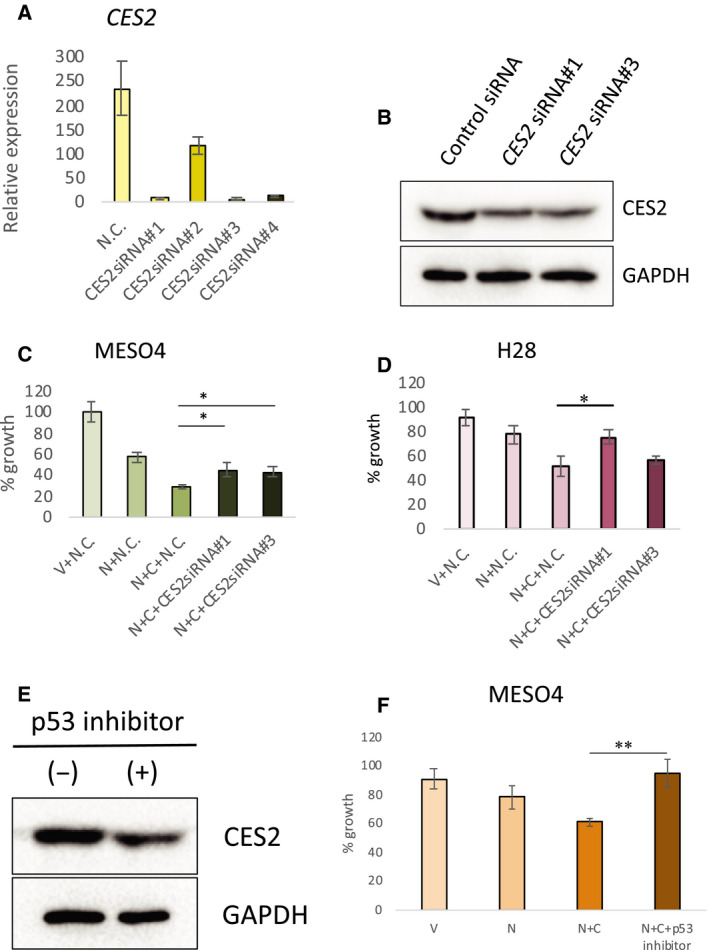
Suppression of the combined effect of nutlin‐3a and CPT‐11 by RNAi for *CES2* or pharmacological inhibition of p53. (A) Validation of *CES2* siRNAs using *CES2*‐overexpressing cells. CES2‐overexpressing ACC‐MESO4 cells (see Fig. 5) were treated with a control double‐stranded RNA (N.C.) or four different siRNAs for *CES2* (CES2siRNA#1, CES2siRNA#2, CES2siRNA#3, and CES2siRNA#4) for 48 h. The level of *CES2* mRNA was examined by qRT‐PCR. The figure shows the mean relative expression level with SEM (*n* = 3). (B) Western blot analysis. MESO4 cells were treated with nutlin‐3a (10 µm) and then with CPT‐11 (20 µg·mL^−1^) and a control siRNA or *CES2* siRNA (CES2siRNA#1 or ‐#3; experimental condition as in C). Protein samples were analyzed by western blotting with anti‐CES2 and anti‐GAPDH antibodies. (C) Suppression of combined efficacy of nutlin‐3a and CPT‐11 by *CES2* siRNA in MESO4 cells. MESO4 cells were treated with vehicle (V) or 10 µm of nutlin‐3a (N) and then treated with a control (N.C.) or *CES2* siRNAs (CES2siRNA#1 and CES2siRNA#3) with or without 20 µg·mL^−1^ of CPT‐11 (C). Cell growth was assessed by the XTT assay. The figure shows the mean percent cell growth with SEM (*n* = 3), relative to vehicle‐treated control cells. Statistical significance was determined by Student's *t*‐test. The * represents *P* < 0.05 between indicated cells. (D) Suppression of combined efficacy of nutlin‐3a and CPT‐11 by *CES2* siRNA in H28 cells. H28 cells were treated and analyzed as in B. The figure shows the mean percent cell growth with SEM (*n* = 3), relative to vehicle‐treated control cells. Statistical significance was determined by Student's *t*‐test. The * represents *P* < 0.05 between indicated cells. (E) Effect of pifithrin‐α (p53 inhibitor) on CES2 expression. MESO4 cells were treated with 10 µm of nutlin‐3a in the presence (+) or absence (−) of 30 µm pifithrin‐α for 48 h. Cell extracts were analyzed by western blotting with anti‐CES2 and anti‐GAPDH antibodies. (F) Suppression of combined efficacy of nutlin‐3a and CPT‐11 by pifithrin‐α (p53 inhibitor) treatment. MESO4 cells were treated with vehicle (V), 10 µm nutlin‐3a (N), 10 µm nutlin‐3a and 20 µg·mL^−1^ CPT‐11 (N + C), 10 µm nutlin‐3a, 20 µg·mL^−1^ CPT‐11, and 30 µm pifithrin‐α (N + C + p53 inhibitor) for 48 h. Cell growth was assessed by the XTT assay. The figure shows the mean percent cell growth with SEM (*n* = 3), relative to vehicle‐treated control cells. Statistical significance was determined by Student's *t*‐test. The ** represents *P* < 0.01 between indicated cells.

**Fig. 5:**
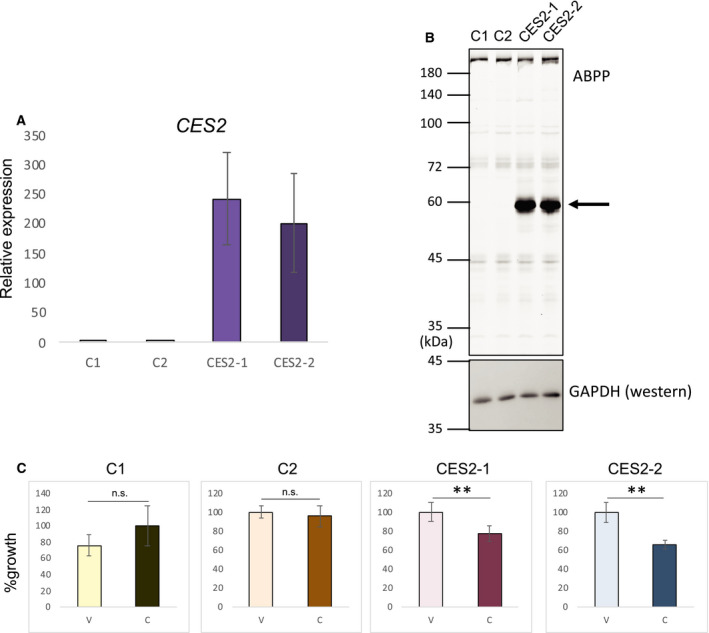
Enhanced efficacy of CPT‐11 in CES2‐overexpressing cells. (A) Expression of *CES2* in established cell lines. The level of *CES* mRNA in two empty vector‐carrying (C1 and C2) and two *CES2*‐overexpressing MESO4 cells (CES2‐1 and CES2‐2) was examined by qRT‐PCR. The figure shows the mean relative expression with SEM (*n* = 3). (B) Detection of CES2 enzyme activity. Protein lysates from control (C1 and C2) and *CES2*‐overexpressing cells (CES2‐1 and CES2‐2) were analyzed by gel‐based ABPP. The arrow indicates the CES2 band. Then, the gel was subjected to western blotting with anti‐GAPDH antibody. The size markers for ABPP are indicated on the left (kD). (C) Cell growth analysis. Control cells (C1 and C2) and CES2‐overexpressing cells were treated for 24 h with vehicle or CPT‐11 (20 µg·mL^−1^) and cell growth was assessed by the XTT assay. The figure shows the mean percent cell growth with SEM (*n* = 3), relative to the vehicle‐treated control cells. Statistical significance was determined by Student's *t*‐test. The ** represents *P* < 0.01. n.s., not significant.

To further document the involvement of *CES2* in the enhanced cell growth inhibition caused by combining CPT‐11 with nutlin‐3a, we inhibited its expression by RNAi (Fig. 4A). The suppression of CES2 protein level was confirmed by western blotting (Fig. 4B). MESO4 cells were treated with two validated siRNAs for *CES2*, resulting in partial suppression of the growth inhibitory effect of CPT‐11 and nutlin‐3a (Fig. 4C). Similarly, the compound efficacy of nutlin‐3a and CPT‐11 was also suppressed by *CES2* siRNAs in H28 cells (Fig. 4D). We also checked the effect of pifithrin‐α, a p53 inhibitor, on *CES2* expression and compound efficacy in MESO4 cells. CES2 expression was inhibited by pifithrin‐α treatment (Fig. 4E). The growth of nutlin‐3a/CPT‐11‐treated cells was fully recovered by pifithrin‐α treatment (Fig. 4F). These results suggest that p53 mediates the compound efficacy of nutlin‐3a and CPT‐11, partially through upregulation of CES2 expression and conversion of CPT‐11 to SN‐38. We also investigated the effect of CPT‐11 alone in MESO4 cells overexpressing CES2. First, we confirmed that increased amounts of *CES2* mRNA were present in two independently established CES2‐overexpressing cell lines (Fig. 5A) and that its enzymatic activity as assessed by gel‐based activity‐based protein profiling (ABPP) method was also increased in these cells (Fig. 5B). On treatment with CPT‐11 alone, we found that the CES2‐overexpressing cells exhibited greater growth suppression than the control cell lines (Fig. 5C). Thus, the expression of CES2 alone sensitized MESO4 cells to CPT‐11 without nutlin‐3a treatment. These results from the experiments with modified CES2 expression indicate that the combinatorial efficacy of CPT‐11 and nutlin‐3a is achieved, at least in part, through the induction of CES2 expression.

## Discussion

Many anticancer drugs are administered as prodrugs and are converted to their chemically active form after being catalyzed by drug‐metabolizing enzymes in the body. Accordingly, the cellular and tissue distribution of such enzymes may affect the efficacy of anticancer prodrugs. CPT‐11 is one of the most commonly used anticancer prodrugs and functions by way of its conversion by CES2 to SN‐38, a Topo I inhibitory molecule [11]. Although it is accepted that the liver is the major site for CPT‐11 activation, increasing evidence suggests a contribution of other CES2‐expressing tissues as well. For example, CES2 is most highly expressed in the intestine and colon, which may be a cause of the severe diarrhea that is one of the major side effects of CPT‐11. Therefore, if cancer cells themselves actively express CES2, the efficacy of CPT‐11 may be increased by conversion within the tumor itself [16, 30]. However, CES2 expression is downregulated in many types of cancer [17, 31, 32, 33].

There are many reports concerning genotoxic stress‐induced p53 activation [34]. Upon severe genotoxic stress caused by 5‐fluorouracil, the expression of *CES2* is activated through p53‐dependent regulation [15, 16]. In our experiments, MM cells also exhibited Dox‐induced upregulation of *CES2* expression. Thus, by combining certain anticancer drugs with others able to induce *CES2*, the efficacy of CES2‐dependent prodrugs might be enhanced in MMs [35]. Given the substantial induction of CES2 protein by Dox in MM cells, the efficacy of the combination of Dox and CPT‐11 in MMs should be studied in future. There are a number of ongoing phase II clinical trials testing CPT‐11 and pegylated Dox (https://clinicaltrials.gov). The combination of Dox and CPT‐11 might be another option for treatment of certain‐types of MM.

Multiple genotoxic drugs, however, tend to cause more adverse events even when a sequential administration protocol is utilized [14, 35]. One possible option to induce *CES2* while avoiding severe adverse and genotoxic effects is to intervene in transcriptional regulation by a molecularly targeted approach. Thus far, regulatory mechanisms controlling *CES2* expression in MM cells have not been determined. We report here, for the first time, that *CES2* expression is regulated by p53 in MM cell lines as revealed by nutlin‐3a treatment. Moreover, the growth inhibitory effect of CPT‐11 was enhanced by nutlin‐3a in H28, 211H, and MESO4 cells. p53 activation by molecular activators such as nutlin‐3a, together with simultaneous treatment with CPT‐11, might therefore represent an effective treatment in MMs expressing wild‐type p53. Because *TP53* mutations can be present but are found only in a fraction of MMs [23, 24], we suggest that those retaining wild‐type *TP53* could be treated with a combination of CPT‐11 and nutlin‐3a. Although adverse events caused by the p53 activator itself need to be considered, severe side effects caused by multiple genotoxic anticancer drugs could be avoided in certain cases. Also for other prodrug substrates of CES2, similar dual administration together with p53 activators may be effective treatments avoiding severe adverse events.

In our analyses, only the partial reversion of the combinatorial effect of CPT‐11 together with nutlin‐3a was observed using RNAi for *CES2*. When the cells were treated with both nutlin‐3a and SN‐38, the active compound of CPT‐11, cell growth was drastically suppressed (Fig. 2F). As shown previously [36, 37], SN‐38 alone had a genotoxic effect strong enough to induce accumulation and activation of p53, despite a much smaller concentration as compared to CPT‐11 (Fig. 2G), whereas CPT‐11 alone did not induce apparent p53 accumulation under these conditions (Fig. 2D). This indicates that nutlin‐3a can boost the anticancer effect of genotoxic drugs. Given the residual CES2 activity in the case of incomplete suppression (Fig. 4), a substantial level of SN‐38 must have been produced and contributed to growth inhibition. Alternatively, CPT‐11 itself potentially contributes to the synergistic effect. NMR analysis revealed that CPT‐11 directly binds to MDM2 and blocks MDM2‐p53 interaction [38]. As speculated, CPT‐11 alone induced a slight, but not negligible, level of p53 accumulation in H28 and 211H cells (Fig. 3B). Taken together, we speculate that the synergistic effect of the compound treatment with nutlin‐3a and CPT‐11 is not limited to upregulation of CES2 and enhanced conversion of CPT‐11 to SN‐38.

The details of the mechanism of *CES2* upregulation by p53 in MMs are still unclear. Several pathways and transcription factors have been implicated in the regulation of *CES2* in human and rodent cells and tissues [39]. Our previous study in colorectal cancer indicated that *CES2* was suppressed by some unknown mechanism not related to *TP53* mutation [17]. We also observed that agonistic agents for the Pregnane X receptor, a candidate for the transcription factor of *CES2* in human hepatoma cells, did not stimulate *CES2* expression in colon cancer cells [17]. In the present study, we observed different induction profiles among known p53 target genes by nutlin‐3a and anticancer drugs in MMs. Although p53 is one of the core transcription factors, there might be various gene‐specific as well as tissue‐specific aspects in the regulation of *CES2* and other p53 target genes. Knowledge of such specificities is important to establish precision cancer medicine.

In conclusion, we found that p53 activators such as nutlin‐3a enhanced antitumor effect of CPT‐11 in MM cells by upregulating the expression of CES2, an enzyme that converts CPT‐11 to its active metabolite SN‐38. Further studies will facilitate an application of the compound use of p53 activators and CPT‐11 to the therapy of MMs.

## Conflict of interest

The authors declare no conflict of interest.

## Author contributions

BH, H‐CL‐O, and TK designed the study; BH, H‐CL‐O, TK, MI, KN, TT, and KK performed the experiments and prepared the figures; BH, TK, HO, TY, OH, and H‐CL‐O contributed to drafting the manuscript. All authors read and approved the final manuscript.

## Data Availability

The datasets used and/or analyzed during the current study are available from the corresponding author upon reasonable request.
